# A digital transformation for primary health care

**DOI:** 10.2471/BLT.23.290726

**Published:** 2024-01-01

**Authors:** Jarbas Barbosa da Silva, Marcos Espinal, Sebastian Garcia-Saiso, James Fitzgerald, Myrna Marti, Ernesto Bascolo, Ana Estela Haddad, Marcelo D’Agostino

**Affiliations:** a Pan American Health Organization, Washington, DC, United States of America (USA).; b Department of Evidence and Intelligence for Action in Health, Pan American Health Organization, Washington, DC, USA.; c Department of Health Systems and Services, Pan American Health Organization, Washington, DC, USA.; d Ministry of Health, Brasilia, Brazil.

The Declaration of Alma-Ata of 1978 confirmed primary health care as essential for the achievement of the goal of health for all.^1^ The declaration marked a paradigm shift in the global approach to health through focusing primary health care on the needs of communities and on tackling health inequities.^2^ To build resilient national health systems based on renewed and strengthened primary health care, information systems for health need improvement and the use of technologies such as telemedicine need expanding in pursuit of the digital transformation of the health sector.^3^

The coronavirus disease 2019 (COVID-19) pandemic has acted as a catalyst and accelerator for the use of information and communication technologies in the health sector, highlighting the need for the digital transformation of this sector. Digital transformation emerges as an opportunity to strengthen and transform health-care models, empowering users within a renewed primary health care model. Moreover, addressing issues related to both noncommunicable diseases and communicable diseases, including endemic and epidemic conditions, is imperative. Doing so requires the establishment of resilient health systems that respond effectively to outbreaks and pandemics and address the challenges posed by diseases, along with the essential aspects of prevention such as immunization and maternal and child health. Mental health, often overlooked, is also key to overall well-being and should be included in the health services supported by digital transformation.

By strengthening information systems for health^4^ and implementing cyber-secure, ethical and interoperable digital solutions,^5^ governments can improve the quality of, and access to, health services. This approach also empowers patients and providers, facilitating continuity of care. Additionally, digital transformation offers opportunities to enhance self-care, promote health and well-being, improve multidisciplinary collaboration among health-care professionals and address inequities in access to health. A comprehensive digital transformation has the potential to improve health outcomes for all, allowing for a healthier population and more sustainable future.^6^^–^^8^

The digital transformation in the health sector is not only about how to use information and communication technologies as supporting tools, or about technological modernization alone. Digital transformation is a cultural change that must consider new health-care models, process reengineering, systems reorganization, and a deeper understanding of people’s behaviour and digital skills. Likewise, such transformation requires a new multisectoral and interdisciplinary approach in the development and implementation of public policies, regulatory frameworks and national digital literacy programmes.

The power of digital transformation has emerged as an innovative and hopeful opportunity to strengthen primary health care. Leveraging technology can support the provision of accessible, efficient and equitable health-care services for all. Digital transformation marks a new era in primary health care that empowers patients and communities through better access to care and information; reduces waiting lists and costs; enables health services to reach the most vulnerable populations; supports collaborative interprofessional practice; and facilitates access to health or the community in general.^9^

To move forward, policy-makers need to embrace digital transformation. Accelerating the implementation of strong primary health care means rethinking and taking advantage of a digitally interdependent and interconnected society.^10^ To do so, governments need to modernize or reformulate public policies, strategies and action plans that optimize the organization of health services; leverage the power of telehealth (which includes telemedicine and tele-education in health) within integrated health services networks; increase digital literacy of the health workforce, providers and patients; and advance in the adoption of new concepts such as connectivity and bandwidth as new determinants of health. Greater investment will be required in the co-creation of digital public goods that allow universal access to electronic medical records, as well as cross-border interoperability processes; digital literacy from early years of schooling; and the development and adoption of regulatory frameworks on the use of open artificial intelligence algorithms, among others.^11^^,^^12^ To reach these goals, greater advocacy, positioning and leadership of the health sector is needed within the framework of digital transformation of governments' initiatives.

The digital transformation of the health sector, particularly in primary health care, requires a human rights approach that ensures universal access to health and universal health coverage. Addressing the prevalent digital divide is essential, giving attention to vulnerable populations and considering principles of gender, equity and ethnicity. Health equity encompasses providing equal opportunities to all individuals, while striving to eliminate discriminatory barriers in health care. By embracing digital solutions with a focus on inclusivity, ethics and cybersecurity, the global health community and governments can revolutionize healthcare models, empower patients and providers, and create resilient health systems capable of effectively preventing and tackling diseases and promoting well-being for all.
